# A conceptual cellular interaction model of left ventricular remodelling post-MI: dynamic network with exit-entry competition strategy

**DOI:** 10.1186/1752-0509-4-S1-S5

**Published:** 2010-05-28

**Authors:** Yunji Wang, Hai-Chao Han, Jack Y Yang, Merry L Lindsey, Yufang Jin

**Affiliations:** 1 Department of Electrical and Computer Engineering, University of Texas at San Antonio, San Antonio, USA; 2 Department of Mechanical Engineering, University of Texas at San Antonio, San Antonio, USA; 3 Center for Research in Biological Systems, University of California at San Diego, La Jolla, California 92093-0043, USA; 4 Department of Medicine, University of Texas Health Science Center at San Antonio, San Antonio, USA

## Abstract

**Background:**

Progressive remodelling of the left ventricle (LV) following myocardial infarction (MI) is an outcome of spatial-temporal cellular interactions among different cell types that leads to heart failure for a significant number of patients. Cellular populations demonstrate temporal profiles of flux post-MI. However, little is known about the relationship between cell populations and the interaction strength among cells post-MI. The objective of this study was to establish a conceptual cellular interaction model based on a recently established graph network to describe the interaction between two types of cells.

**Results:**

We performed stability analysis to investigate the effects of the interaction strengths, the initial status, and the number of links between cells on the cellular population in the dynamic network. Our analysis generated a set of conditions on interaction strength, structure of the network, and initial status of the network to predict the evolutionary profiles of the network. Computer simulations of our conceptual model verified our analysis.

**Conclusions:**

Our study introduces a dynamic network to model cellular interactions between two different cell types which can be used to model the cellular population changes post-MI. The results on stability analysis can be used as a tool to predict the responses of particular cell populations.

## Background

Progressive remodelling of the left ventricle (LV) following myocardial infarction (MI) involves spatial-temporal cellular interactions among different cell types [1]. Apoptosis of myocytes, infiltration of neutrophils, activation of macrophages, activation of endothelial cells, and proliferation of fibroblasts are LV remodelling components [2-5]. These events are accompanied with a temporal flux in the cellular population profiles post-MI [6-13]. Among these cells, macrophages play a pivotal role by coordinating phagocytosis of cellular debris at the MI site and secreting cytokines interleukin-1β, interleukin-6, and tumor necrosis factor α, matrix metalloproteinases (MMPs), tissue inhibitor of metalloproteinases (TIMPs), and growth factors [14-17]. Macrophages are known to undergo a classical activation characterized by pro-inflammatory gene expression in the early stage post-MI. In the later stage post-MI, macrophages undergo an alternative activation characterized by the secretion of factors that promote fibrosis, would healing, neovascularization and granuloma formation. While research has been carried out to investigate the populations of macrophages through different activation schemes [18], the relationship and interactions between these two activated macrophage cellular populations post-MI remains unclear. 

Macrophages are believed to first undergo classical activation, and then proceed through the alternative activation pathway [19,20]. Macrophages do not die locally in the scar tissue, but emigrate from scar tissue to the lymph node system [21]. Thus, the MI site behaves as a network that regulates the exit and entry of macrophages, and the local cytokine environment determines the populations of classically and alternatively activated macrophages. Accordingly, the purpose of this study was to investigate the mathematical relationship among macrophage populations and interactions in a dynamic network. 

The evolution of a dynamic network has been carried out in game theory, social networks, and other biological systems [22-26]. Existing studies have demonstrated that outcomes of tumor growth are determined by the cellular interactions, and these interactions include both cooperation and competition among these cells through a dynamic network [27]. In our research, we have generated stability conditions of a LV network containing two types of macrophages and introduced a new approach to model the temporal activation of macrophages post-MI.

## Results

We developed a dynamic network including two types of macrophages based on a previous graphic model published by Nowak and colleagues [28]. To elucidate the underlying mechanisms of the dynamical evolution, theoretical analysis was carried out and conditions for different evolutionary profiles were obtained. Computer simulations illustrated the dynamic evolution of the network with interactions among two types of macrophages.

### Mathematical model of exit-entry updating law in a dynamic network

**Figure 1:**
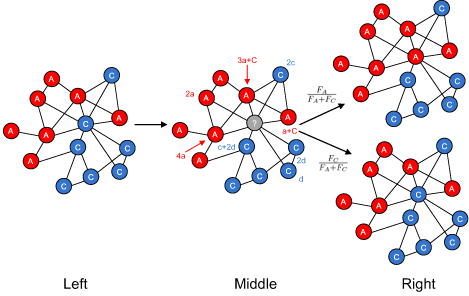
**The structure of the dynamic network in the exit-entry updating process** During the exit-entry updating process, a vacated vertex is replaced by a new macrophage according to the fitness function determined by its neighbouring cells. An original network is shown in the left part of Figure 1 (Left). If a cell, marked in gray, exits the network, the possibility of replacing this cell with either a type *C* (classically activated macrophage) or type *A* (alternatively activated macrophage) cell is determined by the fitness function of the neighbouring cells. Cost function of each cell linked to the vacated cell is shown in the middle of the figure (Middle). In this case, the fitness function of all type *C* cells is calculated as *F_A_* = 4(1 − *ω*)+ *ω*(10*a* + 2*b*). Similarly, the fitness function determined by the connected type *C* cells can be calculated as *F_C_* = 4(1 − *ω*)+ *ω*(3*c* + 5*d*). The gray vertex may be replaced by a type *A* cell with probability , or a type *C* cell with probability of  according to the exit-entry evolutionary strategy, which is shown in the right part of Figure 1 (Right).

A total of N well-mixed cells are distributed over the network. Each cell occupies a vertex of the structured network and links to k other adjacent cells. A linkage between two cells is the edge of the network, denoting the interaction strength between cells. A general interaction matrix can be written as 

where *A* and *C* denote the type of cells in the network (*A* is the alternative activated macrophage and *C* is the classical activated macrophage), parameters *a, b, c, and d* denote the interaction strength between type *A* and *C.* Cells. Specifically, a type *A* cell provides energy *a* to an interacted type *A* cell and provides energy *b* to an interacted type *C* cell. A type *C* cell provides energy *c* to an interacted type *A* cell and *d* to an interacted type *C* cell. In the interaction matrix *I*, different parameter settings of *a, b, c, and d* represent different interaction strengths among cells. Within the network, each cell has an energy function *ε* based on the interactions with all of its linked cells as shown in Figure 1. Fitness function of a cell, *F*, is determined by equation

F = 1 – *ω + ωε*

(1)

where *ω* is a variable between (0, 1), denoting selection strength. The larger the intensity of selection is, the larger the contribution of payoff to the fitness function is. A strong selection is indicated as *ω* = 1 and a weak selection is indicated as *ω* << 1.

In this study, an exit-entry strategy was chosen for the conceptual model, since exit-entry is a fundamental cellular migration scheme for cellular interaction post-MI. In the exit-entry strategy, each iterative step in the exit-entry evolutionary process is called a generation. During the evolutionary process, a cell is chosen randomly to exit in each generation. Assuming a vacated vertex caused by cellular exiting will be only replaced with either a new type *A* or type *C* cell, a probability of replacing by a type *A* cell is determined by *F_A_*/(*F_A_*+*F_C_*), where *F_A_* and *F_C_* are fitness functions of all adjacent cells linked to the vacated vertex. To be specific, the fitness function contributed by all the neighbouring type *A* cells connected to the exiting cell, is calculated as , where *ω* is the intensity of selection, *K_A_* is the number of type *A* cells linked to the exiting cell.

For phenotypes with weak selection, the primary differential equations were set up as 

,   (2)

,

,

,	  (3)

,

	  (4)

	  (5)

where *O*(•) denotes higher order terms of a variable. 

We define *P_A_* and *P_C_* as the ratio of type *A* and *C* cellular population over the total population. Variables *P_AA_*, *P_AC_*, *P_CA_*, *andP_CC_* denote the frequencies of edges between *AA*, *AC*, *CA*, *and CC* interactive cellular pairs. In addition, let *q_X|Y_* denote the conditional probability of a cell type X given the adjacent vertex as cell type Y, where X and Y represent cellular type of *A* or *C*. According to the described exit-entry strategy and the physical meaning of the defined variables, the following identities hold in the structured dynamic network

   (6)

Since *P_AA_*=*q_A|A_P_A_*, *P_A_ and q_A|A_* are two independent variables, equations (2), (3), and (5) are chosen to describe the evolution of the network. 

### Theoretical analysis

In the case of weak selection, *ω* << 1 holds. Therefore, equation (5) represents a fast manifold and equation (2) represents a slow manifold of the dynamics. Our analysis has led to three equilibriums, *P_A_* = 0, 1, *or * of the dynamic network based on the constraints of the interaction strength parameters *a*, *b*, *c*, *d*, and the number of links k in the situation of weak selection. We summarized the following conditions for the three equilibriums.

Case 1: Stable equilibrium at *P_A_* = 1 

A stable equilibrium, *P_A_* = 1, of the system exists if the interaction strength satisfies the following conditions, 

	  (7)

where *P*_*A*0_ is the initial position of *P_A_*.

Case 2: Stable equilibrium at *P_A_* = 0 exists while the conditions shown in equation (8) are satisfied. 

	  (8)

Case 3: Stable equilibrium of *P_A_* ∈ (0, 1) exists with the constraints on interaction strength and the number of links k satisfies the condition

.	  (9)

### Computational simulations

Based on the theoretical analysis, we predicted three types of evolutionary profiles: 1) population of type *A* cells (alternatively activated macrophages) dominates the total cellular population, 2) population of type *C* cells (classically activated macrophages) dominates the total cellular population, or 3) populations of type *A* and type *C* cells reach a dynamic balance in the total cellular population. In addition, the simulations also showed that the evolutionary profiles are related with the interaction strengths, the number of links between cells, and the initial status of the cellular population. To verify the prediction, we designed computer simulations based on the conditions given from equations (7-9) to explore the evolutionary profiles of the network.

### Effects of the interaction strengths on the evolutionary profiles

**Figure 2:**
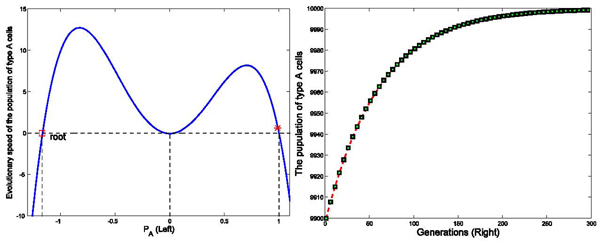
**Effects of interaction strengths on the stability of the dynamic network with different interaction matrix (*P_A_* → 1)** Figure 2 demonstrates the evolutionary dynamics of a network with interaction matrix [1 0; 0 1], k=4, and initial populations of type *A* and type *C* cells are set as 9900 and 100 in a total population of 10000 cells, based on previously published experimental results. The intensity of selection *ω* equals to 0.01. In the left part of Figure 2 (Left), X axis represents the variable *P_A_*, the ratio of type *A* cellular population over the total population. The Y axis represents the evolutionary rate of type *A* cellular population denoted as *Ṗ_A_*. The red star is the initial status of *P_A_*. At the initial status, *Ṗ_A_* is positive, making *P_A_* increase until *P_A_* reaches 1 where *Ṗ_A_* decreases to 0. In the right part of Figure 2 (Right), the simulation results demonstrate that population of type *A* cells increases until it dominates the whole population within 300 generations.

**Figure 3:**
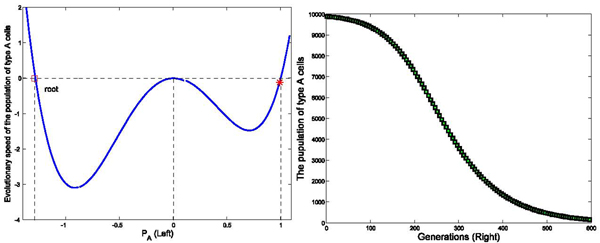
**Effects of interaction strengths on the stability of the dynamic network with different interaction matrix (*P_A_* → 1)** Figure 3 demonstrates the evolutionary dynamics with interaction matrix as [0 -0.5; 1 0], k=4, and initial populations of type *A* and *C* cells are set as 9900 and 100 in a total population of 10000 cells. The intensity of selection *ω* equals to 0.01. As shown in the left part of figure (Left), *Ṗ_A_* is negative in the region of *P_A_* ∈ (0, 1). It means the population of type *A* cells decreases in the interval of *P_A_* ∈ (0, 1) until all type *A* cells exit the system, and *Ṗ_A_* = 0. In the right part of the figure (Right), the simulation results demonstrate that population of type *A* cells decreases until all type *A* cells exit the system within 600 generations.

**Figure 4:**
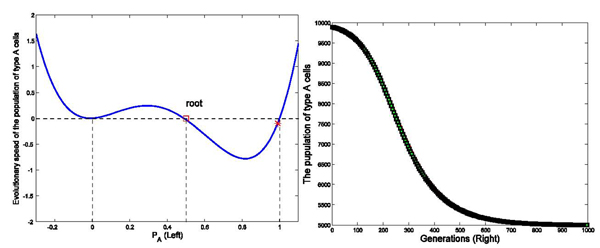
**Effects of interaction strength on the stability of the dynamic network with different interaction matrix (*P_A_* ∈ (0, 1))** Figure 4 shows the evolutionary dynamics with interaction matrix as [0 1; 1 0], k=4, and initial populations of type *A* and *C* cells are set as 9900 and 100 in a total population of 10000, respectively. The intensity of selection *ω* equals to 0.01. Variable *Ṗ_A_* is negative when the initial status of *P_A_* stays in the region between the marked root (red square) and 1 in the left part of the figure(Left). When *P_A_* goes to the marked root, *Ṗ_A_* reaches 0. Accordingly, the population of type *A* cells decreases in the interval of [root, 1] until *P_A_* goes to the root denoted at 0.5 in the simulation. The simulation results shown in the right part of the figure (Right) demonstrate that the population of type *A* cells approached 5000 within 1000 generations.

We have run three sets of computer simulations with interaction matrix in the form of . The number of links between two cells was set as *k* = 4, and the initial population of type *A* and type *C* cells were set as 9900 and 100, respectively. These values were selected based on calculations of the amount of classically versus alternatively activated macrophages at day 3 post-MI. The simulation results demonstrated a dominant population of type *A* cells (*P_A_* → 1) with interaction matrix form *I*_1_ in Figure 2, a dominant population with type *C* cells (*P_A_* → 0) with interaction matrix form *I*_2_ in Figure 3, and a balanced population of both type *A* and type *C* cells (*P_A_* → 0.5) with interaction matrix *I*_3_ in Figure 4. The domination was achieved within finite generations. These 3 sets of simulations demonstrated three different profiles while sharing the same initial status and the number of links among cells, indicating strong interactions among type *A* and type *C* cells to drive the network deviating from the initial dominant type *A* population in these three simulations. These results have been shown accompanying with the analysis on the evolutionary speed of type *A* cellular population. 

### Effects of initial status on the evolutionary profiles of a dynamic network

**Figure 5:**
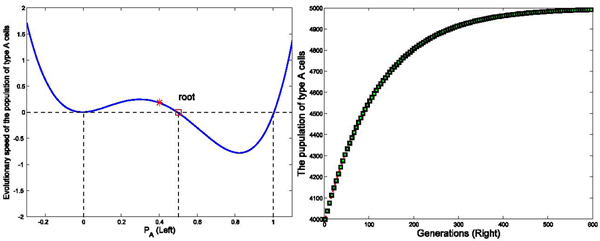
**Effects of initial status on the stability of the dynamic network with different interaction matrix (*P_A_* ∈ (0, 1))** Figure 5 shows the evolutionary dynamics with interaction matrix as [0 1; 1 0], k=4, and initial populations of A and B are as 4000 and 6000 in a total population of 10000 cells. The intensity of selection  equals to 0.01. *Ṗ_A_* is positive when the initial status of *P_A_* stays between 0 and the marked root (red square) in the left part of figure (Left). Accordingly, the population of type *A* cells increases from the initial status in the interval of [0, root] until *P_A_* goes to the root denoted at 0.5 in this simulation. The simulation results shown in the right part of Figure 5 (Right) demonstrate that the population of type *A* cells approaches 5000 within 800 generations.

To investigate the effects of the initial status on the evolutionary profiles, we run three more computer simulations. While sharing the same interaction matrix, the number of links, initial status of *P_A_* was set to 0.99 and 0.4 in the simulations shown in Figures 4 and 5, respectively. In these two simulations, population of type *A* cells and type *C* cells reach a dynamic balance despite the significant differences in the initial status. 

**Figure 6:**
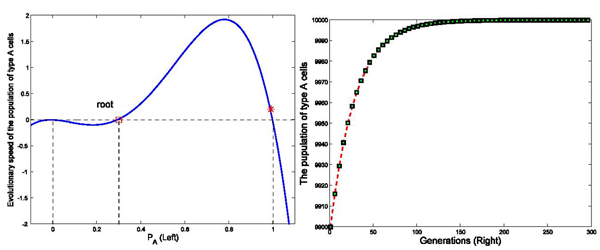
**Effects of initial status on the stability of the dynamic network** Figure 6 shows the evolutionary dynamics with interaction matrix as [0 -1; -2 0], k=4, and initial populations of A and B are set as 9900 and100, respectively, in a total population of 10000. The intensity of selection *ω* equals to 0.01. As shown in the left part of Figure 6, in the case that initial status of *P_A_* stays between the root and 1, *Ṗ_A_* is positive. Therefore, *P_A_* increases from the initial status until it goes to 1 and *Ṗ_A_* goes to 0. Simulation results demonstrate that population of type *A* cells reach 10000 within 300 generations as shown the right part of Figure 6 (Right).

**Figure 7:**
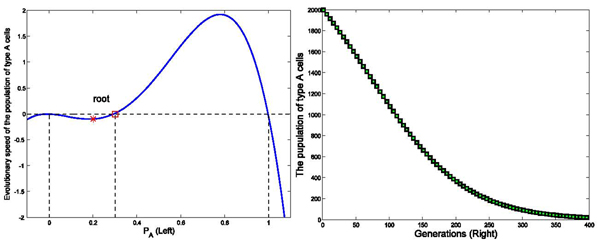
**Effects of initial status on the stability of the dynamic network** Figure 7 shows the evolutionary dynamics with interaction matrix as [0 -1; -2 0], k=4, and initial populations of A and B are set as 2000 and 8000, respectively, in a total population of 10000. The intensity of selection *ω* equals to 0.01. As shown in the left part of Figure 7, in the case that initial status of *P_A_* stays between 0 and the root (red square), *Ṗ_A_* is negative. Thus, *P_A_* decreases from the initial status until it goes to 0 and *Ṗ_A_* goes to 0. Simulation results demonstrate the type *A* cells totally exit the system within 400 generations as shown the right part of Figure 7 (Right).

In simulation pairs shown in Figures 6 and 7, the simulations shared the same interaction matrices (0 -1; -2 0), the number of link (k=4), and the selection strength *ω* = 0.01, but initial status *P_A_* = 0.99 led to a dominant population of type *A* cells in figure 6, and *P_A_* = 0.2 led to a dominant population of type *C* cells in figure 7. Comparisons of these simulations demonstrated that initial status, together with interaction strengths determine the evolutionary profiles of the network. 

### Effects of the number of links on the evolutionary profiles of a dynamic network

**Figure 8:**
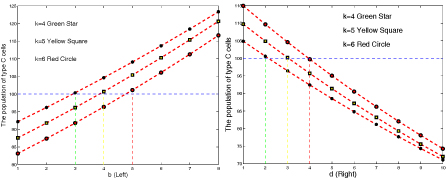
**Effects of number of links on the stability of the dynamic network** Figure 8 shows evolutionary profiles of two dynamic networks with exit-entry interaction matrix [0 b; 1 0] (Left) and [1 0; 0 d] (Right) were simulated with different number of links (k=4, Green stars, k=5 Yellow square, and k=6 Red circles. Population of initial type *C* cells was set as 100 with a total population of 10,000 cells in the system. The intensity of selection *ω* was set as 0.01. The markers are the average populations of type *C* cells after five generations over 10^4^ runs. In the left part of the figure (Left), the horizontal coordinate represents the changes of b value and the vertical coordinate represents the population of type *C* cells. In the right figure, the horizontal coordinate represents the changes of d value and the vertical coordinate represents the population of type *C* cells.

We also designed computer simulations as shown in Figure 8 to illustrate the effects of the number of links on the evolutionary profiles of a network by perturbing the value of k and parameters b and d in the interaction matrix. Given a fixed number of links k, increasing b or decreasing d benefits the increasing population of type *C* cells. Decreasing b or increasing d will lead to decreasing of type *C* population. In addition, given a fixed parameter b or d, variations of k gave the same trend of evolution but changed evolutionary speed. 

All the initial conditions, interaction strength, and the number of links listed in the simulations satisfied the condition associated with the specified equilibrium. The simulations verified predictions on the evolutionary profiles of the network based on our theoretical analysis. 

## Discussion

We have used a dynamic network model to study the cellular interactions with an exit-entry strategy. Our results demonstrated that evolutionary profiles of a dynamic network could be stabilized at different states by perturbing the interaction strength matrix, the number of links, and initial status of the network. We have quantified conditions for stable states in terms of interaction strengths, the initial status, and the number of links in the network. Our computer simulations verified predictions of our analytic results. While we used an exit-entry strategy presented by game theory [28,29], our stability analysis provided not only the stability property but also the convergence states of the system, which is broader than the previous evaluations [28]. We extended analytical stability to the current analysis methods that quantify results using graph theory [24,26,30,31].

Here we have two remarks of our methods. First, we only considered an exit-entry strategy in a structured dynamic network. The exit-entry strategy was chosen because it was the most fundamental and logical cellular function for an initial investigation of the interactions between populations of classically and alternatively activated macrophages post-MI. There exist other evolutionary strategies such as entry-exit, mutation, and imitation. These strategies will need to be considered and potentially incorporated in future models. Secondly, the structure of the dynamic network is fixed by assuming a weak selection, *ω* << 1, and a constant interaction strength matrix. However, stability analysis of the evolutionary strategies with varying structures has more realistic applications to biological systems and has attracted lots of research interest to game theory recently [22,32]. Stability analysis of dynamic networks with varying structure needs to be included in future research models. 

We provide here the first application of a dynamic network model to describe macrophage interactions. We have obtained explicit conditions that determine interaction strength and have established a structure of the network that allows us to predict the stability and equilibrium of the post-MI dynamic network. Our simulation results confirmed the prediction of the stability and the equilibriums of the network. 

## Conclusions

We used a new approach to model the cellular interactions between macrophage activation types in the post-MI setting. The results on stability analysis can be used as a useful tool to predict the responses of specific cellular populations.

## Methods

### Stability analysis of the exit-entry dynamic network

The established mathematical model in equation (2) and (5) is a high order nonlinear system. In a weak selection, *ω* << 1, equation (5) represents the fast manifold of the evolution and equation (2) represents a slow manifold of the system. Thereby, the equilibrium of *q_A|A_* can be approximated from equation (5) by ignoring *ω* dependent terms as 

.	  (10)

The conditional probabilities can then be rewritten as

.	  (11)

In a weak selection, equations (2-3) can be simplified as 

	  (12)

	  (13)

Stability of *P_A_* is analyzed by choosing a positive definite Lyapunov function as . If derivative of V is negative semi-definite, *P_A_* is Lyapunov stable, and the derivative of V can be written as equation (14).

.	  (14)

Define the parameters *c*_0_ = (*k* + 1)*a* + (*k*^2^ – *k* − 1)*b* – *c* +(*k*^2^ − 1)*d*

*c*_1_ = (*k* + 1)(*k −* 3)*a −*(2*k*^2^ − 2*k* − 3)*b* −(*k*^2^ – *k* − 3)*c* −(*k* + 1)*d*

and *c*_2_ = −(*k* + 1)(*k* − 2)(*a* − *b* − *c* + *d*) in the case of weak selection(*ω* << 1), stability of *P_A_* is determined by checking the sign of polynomial . There exist three equilibriums for  i.e., *p_A_* = 0, 1 or . 

Stability of the system can be checked with 3 cases based on the position of the third root and sign of the parameter *c*_2_. The relations of the three equilibriums have been shown in Figures (2, 3, 4, 5, 6, 7).

## Competing interests

The authors declare that they have no competing interests.

## Authors' contributions

Y.J and M.L.L designed the research; Y.J and Y.W performed computational analysis and simulation. J.Y.Y involved in the analysis and provided useful insights in the application to cellular functions. Y.J, Y.W, H.C.H, and M.L.L analyzed the results and wrote the manuscript.
